# Colorimetric Detection of Dopamine Based on Peroxidase-like Activity of β-CD Functionalized AuNPs

**DOI:** 10.3390/molecules30020423

**Published:** 2025-01-20

**Authors:** Sara Anderson, Hamish Shepherd, Kiran Boggavarapu, Janak Paudyal

**Affiliations:** 1 Department of Chemistry and Biochemistry, University of Colorado, Colorado Springs, CO 80918, USA; 2 Department of Chemistry and Physics, McNeese State University, Lake Charles, LA 70605, USA

**Keywords:** functionalized gold nanoparticles, β-cyclodextrin, dopamine, nanozyme, colorimetric assay, sensor

## Abstract

Catalytically active nanomaterials, or nanozymes, have gained significant attention as alternatives to natural enzymes due to their low cost, ease of preparation, and enhanced stability. Because of easy preparation, excellent biocompatibility, and unique optoelectronic properties, gold nanoparticles (AuNPs) have attracted increasing attention in many fields, including nanozymes. In this work, we demonstrated the applicability of beta-cyclodextrin functionalized gold nanoparticles (β-CD-AuNPs) as enzyme mimics for different substances, including TMB and DA. We found that β-CD-AuNPs can catalyze the H_2_O_2_-mediated oxidation of DA. The dopamine signal-off sensor was developed by taking advantage of the peroxidase-like activity of β-CD-AuNPs towards TMB and DA, where both 3,3′,5,5′-tetramethylbenzidine (TMB) and dopamine (DA) may compete for the binding sites with β-CD-AuNPs. As a result, the presence of dopamine can be detected even through the naked eye (up to the concentration of 3.75 µM) and using a spectrophotometer (up to the concentration of 1.0 µM) by monitoring the disappearance of the blue color of the oxidized form of TMB in the presence of dopamine. Furthermore, no obvious disappearance of color was observed at lower concentrations of interferences including ascorbic and uric acid. Given the versatility of cyclodextrin to host large numbers of analyte molecules, we envision that a similar principle can be applied for the detection of other analyte molecules of biological, medical, and environmental significance.

## 1. Introduction

Catalytically active nanomaterials (nanozymes) are emerging as one of the most promising substitutes for native enzymes, sparking enormous research interest [1]. Their inherent superiorities over natural enzymes, including the ease of synthesis, low costs, long storage time, and better stability, have positioned them as ideal catalysts for mimicking the catalytic function of natural enzymes [2,3]. This potential has led to the rapid discovery, development, and application of novel and highly active nanozymes, making it a lively field of research.

Significant research efforts are being directed into the development of nanozyme catalysts that mimic horseradish peroxidase (HRP), or other peroxidases, among other enzyme mimetics [4,5]. HRP’s efficient catalysis of oxidation reactions has made it a highly sensitive detection tool in various assays, including the enzyme-linked immunosorbent assay (ELISA), immunohistochemistry, and Western blotting. The broad interest in HRP-mimicking catalysts lies in their potential to serve as substitutes for the native enzyme in a wide range of applications, from (bio)sensing and immunoassay to imaging, disease diagnosis, cancer therapy, and environmental monitoring, sparking intrigue about their diverse potential [3,6,7]. Nanomaterials, such as metal [8,9] and metal oxide nanoparticles [10], graphene [11], carbon dots [12], carbon nitrides [12] etc., have been explored for their unique peroxidase-mimetic catalytic activities.

Because of easy preparation, excellent biocompatibility, and their unique optoelectronic properties, gold nanoparticles (AuNPs) have attracted increasing attention in many fields. Historically, gold atoms were considered inert, but, most recently, both supported AuNPs and unsupported colloidal AuNPs have attracted more attention as catalysts and have shown enzyme-mimicking activities, including peroxidase mimetics [13,14,15]. Jv et al. first reported the peroxidase-mimicking activity of AuNPs on cysteine-functionalized positively charged gold nanoparticles (Cys-AuNPs) [16]. They found that the catalytic activity of citrate-coated AuNPs is lower than that of Cys-AuNPs. This shows that surface functionalities may play a critical role besides the obvious active sites of AuNPs, as demonstrated in Liu et al.’s [17] work. Lin and Chen’s groups [18] compared the naked, amino-modified, and citrate-modified AuNPs and found that “naked” AuNPs exhibited superior catalytic activity compared to the other AuNPs. The gold atoms on the surface of AuNPs might contribute to their catalytic activity. When TMB was selected as the substrate, citrate-modified nanozymes exhibited higher peroxidase-like activity than amino-modified AuNPs. This phenomenon was reversed when choosing ABTS as the substrate, indicating the crucial role of both catalyst and substrate and their charge characteristics in defining peroxidase-mimicking activity. The peroxidase-like activity of AuNPs with different functionalities has also been used to detect biothiols [19], ascorbic acid [20], Cr(VI) [21], and bacteria [22].

Peroxidase-like activity has recently been reported in β-CD functionalized AuNPs [20,23] for the H_2_O_2_ catalyzed oxidation of 3,3′,5,5′-tetramethylbenzidine (TMB). The peroxidase-mimetic activity was much higher compared to the citrate-coated AuNPs, even though the size of the citrate-coated AuNPs (~13 nm) was much smaller compared to β-CD-AuNPs (~20 nm). The superior peroxidase-like activity was attributed to the synergistic effects of CD molecules and Au particles. Here, we first simplified the synthesis of β-CD-AuNPs by reducing the synthesis time of β-CD-AuNPs by more than 50% (less than half an hour instead of over an hour). We then demonstrated the general applicability of β-CD-AuNPs as enzyme mimics not only for TMB but also for other substrates, such as OPD and DA. We found that β-CD-AuNPs can also catalyze the H_2_O_2_-mediated oxidation of OPD and DA. By taking advantage of the peroxidase-like activity of β-CD-AuNPs towards TMB and DA, a signal-off dopamine sensor was developed.

Dopamine (DA) is a crucial neurotransmitter in the nervous system, with implications for various disorders such as Parkinson’s disease, dementia, and schizophrenia. Detecting DA is essential for understanding its roles in neurobiology and disease diagnosis [24]. Therefore, developing a simple, cost-effective, and highly sensitive method to detect dopamine is paramount. While various sophisticated techniques, such as electrochemical strategies, chemiluminescence, chromatography, capillary electrophoresis, and spectroscopic approaches, have been successfully employed for dopamine determination, most require expensive equipment and complex operations [25]. In contrast, the colorimetric assay presents a promising solution, offering theoretical and technical simplicity. While colorimetric detection of DA has been achieved based on the surface plasmon resonance of AuNPs [26,27], this work utilizes the peroxidase-mimicking activity of β-CD-AuNPs. The peroxidase-mimicking activity of various nanomaterials and the inhibition of dopamine have been recently exploited for the detection of dopamine using colorimetric [28,29,30] or fluorometric methods [30,31].

In this work, we report the design of a novel colorimetric sensor for dopamine detection based on the peroxidase-like activity of β-CD-AuNPs. By utilizing the β-CD onto the surface of AuNPs, we aim to enhance the selective interaction between dopamine and the β-CD-AuNPs. We demonstrated that β-CD-AuNPs catalyze the oxidation of dopamine to aminochrome by H_2_O_2_, most likely due to an increase in the local concentration of dopamine at the surface of heterogeneous catalysts. We have developed a colorimetric signal-off dopamine sensor based on the competition between dopamine and TMB for possible binding sites at the surface of the β-CD-AuNPs. As a result, the presence of dopamine can be detected even through the naked eye (up to the concentration of 3.75 µM) and using a spectrophotometer (up to the concentration of 1.0 µM) by monitoring the disappearance of the blue color of the oxidized form of TMB in the presence of dopamine. Furthermore, no obvious color change was observed in the presence of lower concentrations of common interferences, including ascorbic acid and uric acid.

## 2. Results and Discussion

### 2.1. Synthesis and Characterization of β-CD-AuNPs

**Figure 1:**
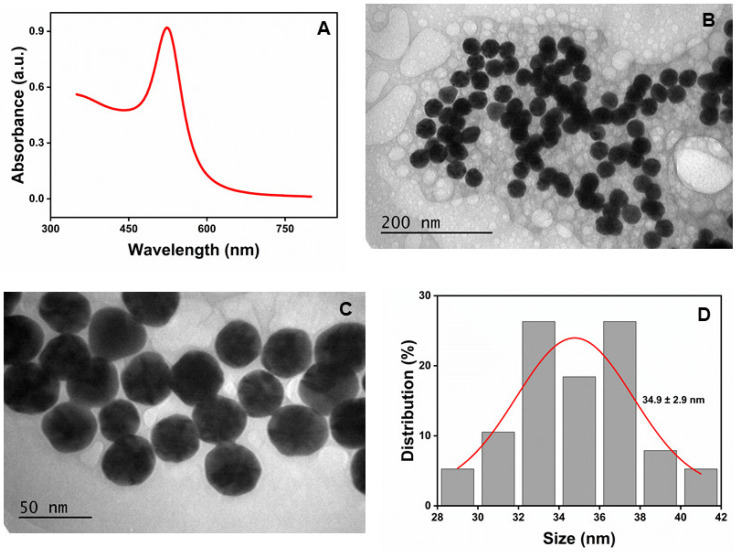
Synthesized β-CD-AuNPs: (**A**) UV–Vis spectroscopy showing a characteristic peak at 524 nm. (**B**,**C**) TEM image of the β-CD-AuNPs with different magnifications and (**D**) histogram showing the size distribution of the synthesized β-CD-AuNPs (n = 38).

As previously reported [23], β-CD-AuNPs were prepared using β-CD as a reducing and stabilizing agent, with slight modification. β-CDs were added to the boiling solution of gold salt (in a 10 mM sodium phosphate buffer at pH 7.0) instead of gradual heating to reduce the Au^3+^ into Au^0^. This straightforward addition of a reducing agent in the boiling solution of gold salt instead of gradually heating the mixture of gold salt and reducing agent reduces the time for the synthesis of β-CD (less than half an hour instead of over an hour). The reduction process leads to the nucleation, growth, and formation of AuNPs. Figure 1A shows the characteristic absorption spectra of the synthesized β-CD-AuNPs, with a sharp SPR band at 524 nm, indicating the typical features of a gold nanosphere. The size of the AuNPs was estimated as ~35 nm based on the absorbance ratio at SPR and 450 nm (1.925), as described in the literature [32]. The concentration of the as-prepared AuNPs was estimated as 0.28 nM using absorption data and a molar absorption coefficient of 3.21 × 10^−9^ M^−1^cm^−1^ as described in the same reference. Figure 1B,C show the high-resolution transmission electron microscopy (TEM) images of the β-CD-AuNPs with different magnifications. TEM revealed the spherical/quasi-spherical shape of β-CD-AuNPs. The size of the AuNPs is 34.9 ± 2.9 nm with a very narrow distribution, as shown in Figure 1D. The size was estimated from TEM images in the ImageJ 1.54 g software.

### 2.2. Characterization of β-CD at the Surface of AuNPs

**Figure 2:**
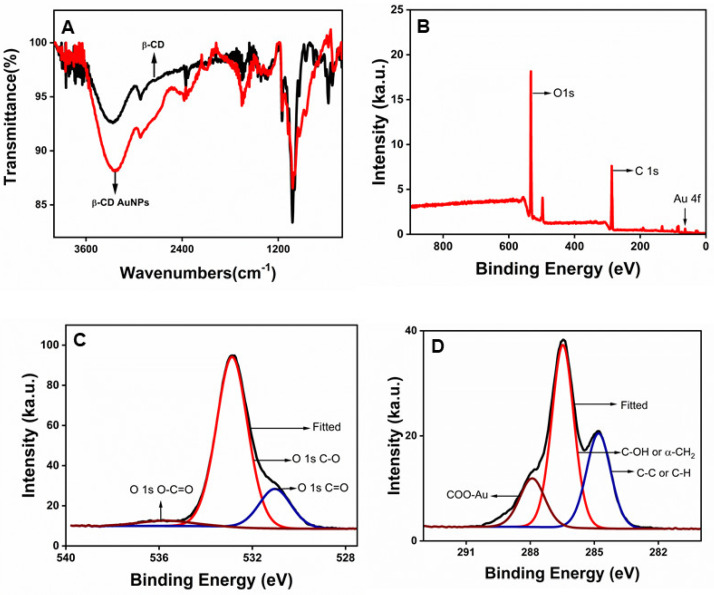
β-CD at the surface of the AuNPs: (**A**) the comparison of Fourier transform infrared (FT-IR) spectra of purified β-CD-capped AuNPs (red) with pure β-CD powder (black), (**B**) an X-ray photoelectron spectroscopy (XPS) scan survey of a wide region showing all the elements present in double-purified AuNPs, (**C**) a high-resolution core level XPS scan of oxygen, and (**D**) a high-resolution core level XPS scan of carbon.

Fourier transform infrared (FT-IR) spectroscopy and X-ray photoelectron spectroscopy (XPS) were used to confirm the presence of beta-cyclodextrin (β-CD) at the surface of the AuNPs. The comparison of the FT-IR spectra of double-purified and dried AuNPs with the pure β-CD-powder, as shown in Figure 2A, shows similar profiles of -OH at ~3354 cm^−1^ and C-O groups at ~1100 cm^−1^, indicating that beta-cyclodextrins are retained at the surface of the β-CD.

X-ray photoelectron spectroscopy (XPS) was further used to probe the surface chemistry of AuNPs. The binding energy (BE) of electrons of an atom provides information about the binding and coordination of the functional groups as it is sensitive to the local electronic state of adjacent atoms. A wide region survey and high-resolution core-level scans of all elements were recorded and calibrated with the C_1s_ 284.8 eV as the reference peak. Figure 2B shows a wide region survey scan showing the presence of Au, C, and O in the sample containing double-purified AuNPs. The high-resolution spectra of the oxygen were further obtained, as shown in Figure 2C. The deconvoluted O 1s spectrum consists of three distinct binding energies at 531.02, 532.87, and 535.73 eV, attributed to C=O, C-O, and O-C=O, respectively [33]. The deconvoluted C 1s spectrum consists of four distinct binding energies at 284.8, 286.47, 287.91, and 289.26 eV, which are attributed to adventitious carbons (C-C or C-H), the hydroxyl (C−OH), and/or the α-carbons (CH_2_), the coordinated carboxylates (COO-Au), and free carboxyl moieties (COOH or COO−), respectively [34,35].

### 2.3. Investigation of Peroxidase-Mimicking Activity of β-CD-AuNPs Using TMB as a Substrate

We first tested the peroxidase-like activity of β-CD-AuNPs using commonly used peroxidase substrate 3,3′,5,5′-tetramethylbenzidine (TMB) in the presence of H_2_O_2_. TMB was chosen due to its higher sensitivity, lowest potential carcinogenicity, better color purity of the colorimetric product, and reasonable stability of the oxidation product as compared to other chromogens [36]. Peroxidases oxidize TMB, a colorless compound, into a blue-colored complex in the presence of H_2_O_2_.

**Figure 3:**
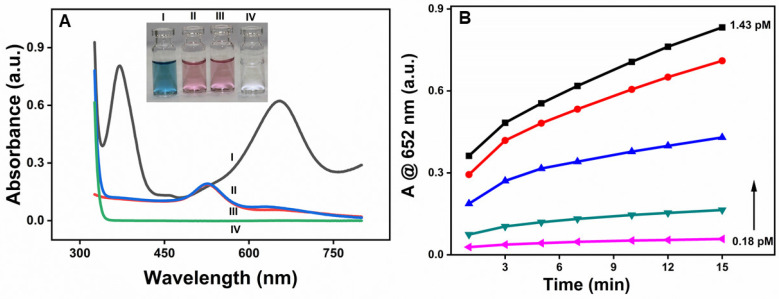
Peroxidase-like activity of β-CD-AuNPs for the oxidation of TMB: (**A**) UV–vis spectrum of the reaction mixture TMB-H_2_O_2_ containing β-CD-AuNPs and other control samples. Inset: Photographs showing the color of the reaction mixture of the TMB-H_2_O_2_ system containing β-CD-AuNPs and other control samples. Curves and samples I to IV represent the TMB + H_2_O_2_ + β-CD-AuNPs, H_2_O_2_ + β-CD-AuNPs, TMB + β-CD-AuNPs, and TMB + H_2_O_2_ respectively. Reaction conditions: 100 µL of 6× concentrated AuNPs (final concentration of 0.72 pM), 12 µL of 20 mM of TMB (final concentration of 0.1 mM), 24 µL of 1M H_2_O_2_ (final concentration of 10 mM) in a reaction mixture containing 10 mM of sodium acetate buffer pH 5.0 and a reaction time of 10 min. (**B**) Evolution of the color change in the TMB-H_2_O_2_ system as observed by the increase in absorbance at 652 nm as a function of time from 1 min to 15 min and as a function of final concentration of β-CD-AuNPs from 0.18 pM, 0.36 pM, 0.54 pM, 0.72 pM, and 1.43 pM, respectively.

As shown in Figure 3A, β-CD-AuNPs oxidize TMB in the presence of H_2_O_2_, as prominent absorption peaks appeared at 370 nm and 652 nm upon the addition of β-CD-AuNPs to the TMB-H_2_O_2_ system due to the oxidation of TMB (Curve I). However, no such peak was obtained without adding β-CD-AuNPs (Curve IV). Similarly, the oxidation peaks of TMB are absent in both the samples containing gold nanoparticles in the presence of H_2_O_2_ only (Curve II) and in the presence of TMB only (Curve III). The observation shows that β-CD-AuNPs can oxidize TMB in the presence of H_2_O_2_ like the natural horseradish peroxidase enzyme. The spectral change can also be monitored through the naked eye, thanks to the blue color of the oxidized form of TMB, as shown in the photograph in the inset of Figure 3A. The color change can easily be differentiated from other control samples—from left to right—the blue color of the reaction mixture of TMB + H_2_O_2_ + β-CD-AuNPs, no color for the reaction mixture of TMB + H_2_O_2_, and the faint red color for the reaction mixtures of β-CD-AuNPs + H_2_O_2_ or β-CD-AuNPs + TMB. Furthermore, Figure 3B shows that the oxidation of TMB increases with the increase in concentration of the gold nanoparticles and with the reaction time, confirming the role of β-CD-AuNPs on the oxidation of TMB. The formation of more products as time increases shows the dynamic process of TMB oxidation by β-CD-AuNPs.

### 2.4. Applicability of β-CD-AuNPs as Peroxidase Mimetics—OPD as a Substrate

Although the TMB-H_2_O_2_ system is widely used to detect peroxidase-like activity, it has been reported that it will be premature to consider any reagent or nanoparticle that changes TMB color in the presence of H_2_O_2_ as a peroxidase-like enzyme or nanozyme [37] as TMB can be oxidized by many other common redox reaction systems, such as solutions containing Fe^3+^ or Cu^2+^. Therefore, we also tested β-CD-AuNPs with other peroxidase substrates such as O-Phenylenediamine (OPD) and dopamine (DA) to confirm the peroxidase-like activity and to make it generally applicable peroxidase mimetics.

**Figure 4:**
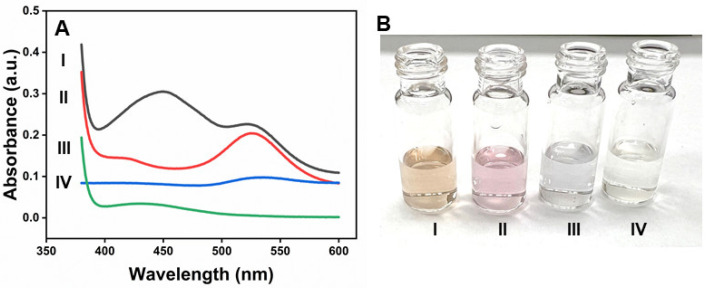
Peroxidase-like activity of β-CD-AuNPs for the oxidation of OPD: (**A**) UV–vis spectrum of the reaction mixture OPD-H_2_O_2_ containing β-CD-AuNPs and other control samples. (**B**) Photographs showing the color of the reaction mixture of the OPD-H_2_O_2_ system containing β-CD-AuNPs and other control samples. For both Figure A and B, I—a mixture of OPD + H_2_O_2_ + β-CD-AuNPs, II—a mixture of H_2_O_2_ + β-CD-AuNPs, III—a mixture of OPD + β-CD-AuNPs, and IV—a mixture of OPD + β-CD-AuNPs. Reaction conditions: 100 µL of 6× concentrated AuNPs (final concentration of 0.72 pM), 24 µL of 100 mM of OPD (final concentration of 1 mM), 24 µL of 10 M of H_2_O_2_ (final concentration of 100 mM) in a reaction mixture containing 10 mM of sodium acetate buffer pH 5.0 and a reaction time of 60 min.

Figure 4A shows that prominent absorption peaks at 450 nm appeared upon the addition of β-CD-AuNPs to the OPD-H_2_O_2_ system due to the oxidation of OPD (Curve I). The naked eye can also monitor the spectral change, thanks to the yellowish color of the oxidized form of OPD-2,3-diaminophenazine (DAP), as shown in the photograph of Figure 4(B-I). However, no such peak or color change was observed without adding β-CD-AuNPs (Curve IV and Figure 4(B-IV)). Similarly, the oxidation peaks of DAP are absent in both the samples containing gold nanoparticles in the presence of H_2_O_2_ only (Curve II) and in the presence of OPD only (Curve III). Furthermore, it is important to note that β-CD-AuNPs are stable in H_2_O_2_, as shown in Curve II, indicating that H_2_O_2_ alone does not destabilize β-CD-AuNPs. The color of β-CD-AuNPs also remains intact, as shown in Figure 4(B-II). However, β-CD-AuNPs aggregates in the presence of OPD in the absence of H_2_O_2_ without undergoing oxidation. The color of β-CD-AuNPs changes into blue due to aggregation, as shown in Figure 4(B-III). This observation indicates that β-CD-AuNPs can oxidize OPD in H_2_O_2_ like the natural horseradish peroxidase enzyme.

### 2.5. Applicability of β-CD-AuNPs as Peroxidase Mimetics—DA as a Substrate

**Figure 5:**
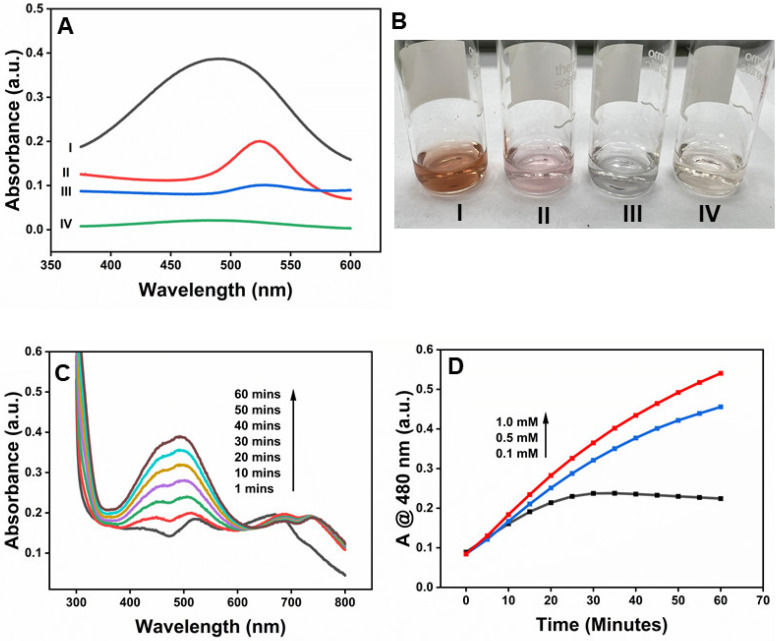
Peroxidase-like activity of β-CD-AuNPs for the oxidation of DA: (**A**) UV–vis spectrum of the reaction mixture DA-H_2_O_2_ containing β-CD-AuNPs and other control samples. (**B**) Photographs showing the color of the reaction mixture of the DA-H_2_O_2_ system containing β-CD-AuNPs and other control samples. For both Figure A and B, I—mixture of DA+ H_2_O_2_ + β-CD-AuNPs, II—mixture of H_2_O_2_ + β-CD-AuNPs, III—mixture of DA + β-CD-AuNPs, and IV—mixture of DA + β-CD-AuNPs Reaction conditions: 100 µL of 6× concentrated AuNPs (final concentration of 0.72 pM), 7.4 µL of 100 mM of DA (final concentration of 0.3 mM), 24 µL of 10 M of H_2_O_2_ (final concentration of 100 mM) in a reaction mixture containing 10 mM of sodium acetate buffer pH 5.0 and a reaction time of 60 min. (**C**) Evolution of the color change in the DA-H_2_O_2_ system as observed by the increase in absorbance at 480 nm as a function of time from 1 min to 60 min and (**D**) the change in absorbance at 480 nm as a function of different concentrations of dopamine.

The fact that HRP oxidizes dopamine in the presence of H_2_O_2_ [38] suggests that the β-CD-AuNPs could, similarly, act as a catalyst for this oxidation reaction of DA into aminochrome. Indeed, we find that β-CD-AuNPs can catalyze the oxidation of DA to aminochrome in the presence of H_2_O_2_. Figure 5A shows that prominent absorption peaks at 480 nm appeared upon the addition of β-CD-AuNPs to the DA-H2O2 system due to the oxidation of DA (Curve I). The spectral change can also be monitored through the naked eye, thanks to the brownish-orange color of the oxidized form of DA-aminochrome or possibly the polymerized form, as shown in the photograph of Figure 5(B-I). However, no such peak or color change was observed without adding β-CD-AuNPs (Curve IV and Figure 5(B-IV)). Similarly, the oxidation peaks of aminochrome are absent in both the samples containing gold nanoparticles in the presence of H_2_O_2_ only (Curve II) and in the presence of DA only (Curve III). Furthermore, it is important to note that β-CD-AuNPs are stable in H_2_O_2_, as shown in Curve II, indicating that H_2_O_2_ alone does not destabilize β-CD-AuNPs. The color of β-CD-AuNPs also remains intact, as shown in Figure 5(B-II). However, as reported previously, β-CD-AuNPs aggregate in the presence of DA and in the absence of H_2_O_2_ without undergoing oxidation [26]. The color of β-CD-AuNPs changes into blue due to aggregation, as shown in Figure 5(B-III). This observation indicates that β-CD-AuNPs can oxidize DA in H_2_O_2_, like the natural horseradish peroxidase enzyme.

Figure 5C shows the evolution of the peak at 480 nm upon the addition of β-CD-AuNPs to the DA-H_2_O_2_ system due to the oxidation of DA. Immediately after the addition of β-CD-AuNPs (at around the 1 min reaction time) in the DA-H_2_O_2_ system, β-CD-AuNPs undergo aggregation, as indicated by the appearance of a peak at around 670 nm. However, with the increase in the reaction time, the peak shifts from ~670 nm to ~690 nm, indicating the formation of the bigger size of the nanocluster. The formation of a bigger nanocluster coincides with the appearance of an aminochrome peak at around 480 nm, indicating the two dynamic processes of β-CD-AuNPs. Interestingly, as the reaction time increases, the peak at 480 nm increases due to the formation of more aminochrome, but the peak at 690 nm decreases. The decrease in intensity at 690 nm is smaller than the increase in peak at 480 nm. It is unclear how the oxidation of dopamine impacts the aggregation state of the β-CD-AuNPs. The detailed mechanism is currently being investigated in the lab.

Figure 5D shows the concentration-dependent oxidation of DA in the presence of a fixed concentration of β-CD-AuNPs as a function of time. At a lower concentration of DA (0.1 mM), absorbance at 480 nm increases with an increase in the reaction time and becomes saturated at around 25 min. At higher concentrations of 0.5 mM and 1 mM, the absorbance increases with an increase in reaction time of up to 60 min. As the concentration of DA increases from 0.1 mM to 1 mM, the rate of formation of aminochrome increases, as indicated by the larger slope. These facts indicate that the signal is, in fact, due to the oxidation of DA by β-CD-AuNPs.

### 2.6. Detection of Dopamine

**Figure 6:**
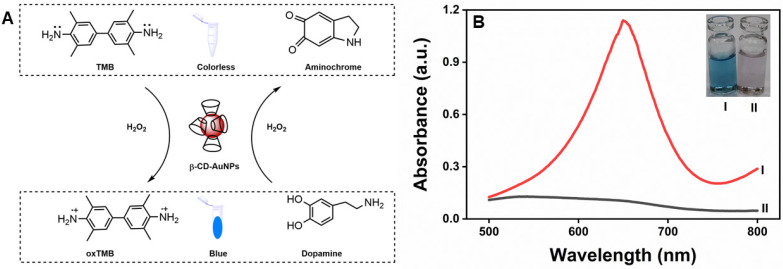
The disappearance of the blue color of the oxidized TMB upon the addition of 100 µM of dopamine: (**A**) Schematic showing that the absorbance profile of the TMB-H_2_O_2_-β-CD-AuNP system collapses upon the addition of the dopamine. (**B**) Visible spectra of TMB-H_2_O_2_-β-CD-AuNP system and the photographs in the inset before (I) and after (II) the addition of the dopamine. Reaction conditions: 100 µL of 6× concentrated AuNPs (final concentration of 0.72 pM), 12 µL of 20 mM of TMB (final concentration of 0.1 mM), 24 µL of 10 M of H_2_O_2_ (final concentration of 100 mM), and a reaction time of 15 min in a reaction mixture containing 10 mM of sodium acetate buffer pH 5.0.

From the above results, it is clear that β-CD-AuNP can oxidize TMB into a blue-colored product and DA into aminochrome in a concentration-dependent manner. Therefore, it is likely that DA and TMB compete for the binding sites with β-CD-AuNPs. We found that the blue color of oxidized TMB reverses upon the addition of dopamine. Based on the reversal of the color of the TMB, we have developed a new dopamine sensor, as depicted in Figure 6A. We found that absorbance at 652 nm, due to the oxidation of TMB in the presence of H2O2 and β-CD-AuNPs, increases with an increase in time and becomes saturated. Upon the addition of 100 µM dopamine, however, the blue color disappears, as observed visually in the inset of Figure 6B, spectroscopically through the collapse of the peak at 652 nm, as shown in Figure 6B.

As reported before, the peroxidase-like activity of β-CD-AuNPs is most likely due to the generation of oxygen-reactive species (ROS) like hydroxyl radicals (·OH) [23]. These hydroxyl radicals generate radical cations, forming a blue-colored charge transfer complex with a second TMB molecule [39]. Upon the addition of dopamine, the blue color of TMB disappears, which is the basis of the colorimetric detection of dopamine, as depicted in Figure 6A. The actual reason for the disappearance of color is being investigated. However, dopamine is likely a scavenger for the hydroxyl free radical [40] generated through β-CD-AuNPs and H_2_O_2_. As demonstrated in Section 2.5, β-CD-AuNPs, indeed, oxidizes dopamine to aminochrome.

To maximize the detection window for the detection of dopamine, we optimize the different factors such as the buffer pH, H_2_O_2_ concentration, TMB concentration, and reaction time. We first explored the dependence of pH values on the TMB-H_2_O_2_ system by measuring the absorbance at 652 nm in a 10 mM acetate buffer at a fixed concentration of the TMB, H_2_O_2_, and the reaction time. The difference in absorbance at 652 nm in the presence and absence of hydrogen peroxide was used to find the optimum pH for the H_2_O_2_-mediated color development process as in the natural HRP. The activity increases with an increase in pH from 3.5 to 5.0, provides the maximum absorbance as well as absorbance difference of 0.38 at pH 5.0, and decreases again with an increase in pH to 5.5 as shown in Figure S1. The activity was not tested in alkaline and neutral mediums as the acidic medium is necessary for the oxidation of TMB, and H_2_O_2_ is known to degrade in an alkaline medium [41]. Therefore, pH 5.0 was used as an optimum buffer pH. We found that absorbance at 652 nm (oxidation of TMB) increases with an increased concentration of AuNPs and TMB, as shown in Figure S2. The rate increases sharply from 0.18 pM to 0.72 pM and slowly afterward. To minimize the use of AuNPs, 0.72 pM (300 µL 6× concentrated) was considered an optimum concentration. We found that the absorbance increases with an increase in TMB concentration in the 0.1 to 0.4 mM range. However, non-specific color formation increases with an increase in the concentration of the TMB. Therefore, a relatively lower concentration of the TMB was used throughout the experiment. The concentration of H_2_O_2_ has a huge impact on the formation of color in the TMB-H_2_O_2_ system; we found that absorbance increases with an increase in H_2_O_2_ and becomes saturated at about 100 mM, as shown in Figure S3A. Finally, the reaction time was optimized, and the absorbance of the TMB-H_2_O_2_ system increased with an increase in the reaction time and was saturated at about 15 min. Therefore, 15 min is considered the optimum reaction time, as shown in Figure S3B.

### 2.7. Calibration Curve for the Detection of Dopamine

**Figure 7:**
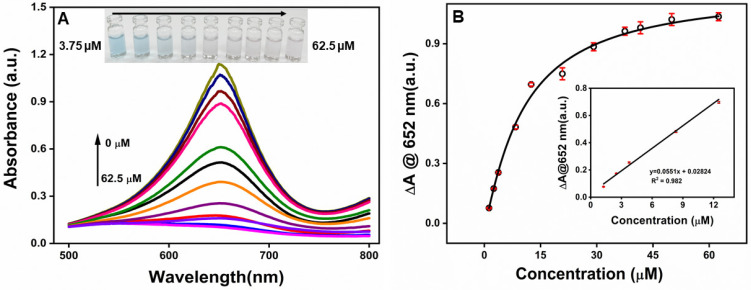
(**A**) Typical absorption spectra of the β-CD-AuNPs −TMB−H_2_O_2_ system in the presence of DA at various concentrations. From top to bottom, 0, 1.25, 2.5, 3.75, 8.34, 12.5, 20.8, 29.1, 37.5, 41.67, 50.0, and 62.5 µM. Inset photographs of the β-CD-AuNPs−TMB−H_2_O_2_ system in the presence of different amounts of DA that can be visualized through the naked eye. (**B**) Plots of the change in absorbance (ΔA) at 652 nm as a function of DA concentration. Inset is the linear calibration plot for DA detection.

Optimized conditions were used to detect dopamine. Figure 7A shows the visible spectrum of the β-CD-AuNPs−TMB-H_2_O_2_ system at different concentrations of dopamine. The absorbance at 652 nm gradually decreases with an increase in the dopamine concentration from 0−62.5 μM of DA. A precise color change from blue to light blue and eventually colorless could be differentiated by the naked eye along with the increase in DA concentration from 3.75 μM to 62.5 μM as shown in the photographs in Figure 7A inset. Figure 7B shows the change in absorbance (ΔA) at 652 nm as a function of dopamine concentration. The value of ΔA increases with an increase in dopamine concentration from 1.25 μM to 62.5 μM. However, it starts to become saturated at a concentration of 37.5 µM. The inset of Figure 7B shows the linear response of the absorbance over the concentration range of 1.25−12.5 μM of DA. The limit of detection was found to be 1.08 μM based on the S/N ratio of three.

Although a lower limit of detection has been achieved [30,42,43] in the previously reported colorimetric sensor based on the peroxidase-like activity of nanomaterial, they usually take a longer incubation time than the current method. Furthermore, reported nanozymes usually involve long and complex synthetic methods as compared to our method. Table S1 provides the overview of the comparison of our method to the reported methods in terms of synthesis and analytical performance. Further understanding the peroxidase-like activity of β-CD-AuNPs, especially the role of the -OH groups, will enhance the detection limit. Compared to the previous peroxidases, which are limited to a specific analyte, our method could potentially be used for detecting other analytes due to the presence of cyclodextrin, which can form an inclusion compound with many analytes [44].

### 2.8. Specificity of the Assay for the Detection of Dopamine

To evaluate the selectivity of this detection platform, possible interfering substances, including leucine, valine, uric acid, aspartic acid, ascorbic acid, and creatine, were tested, and the absorbance at 652 nm was recorded.

**Figure 8:**
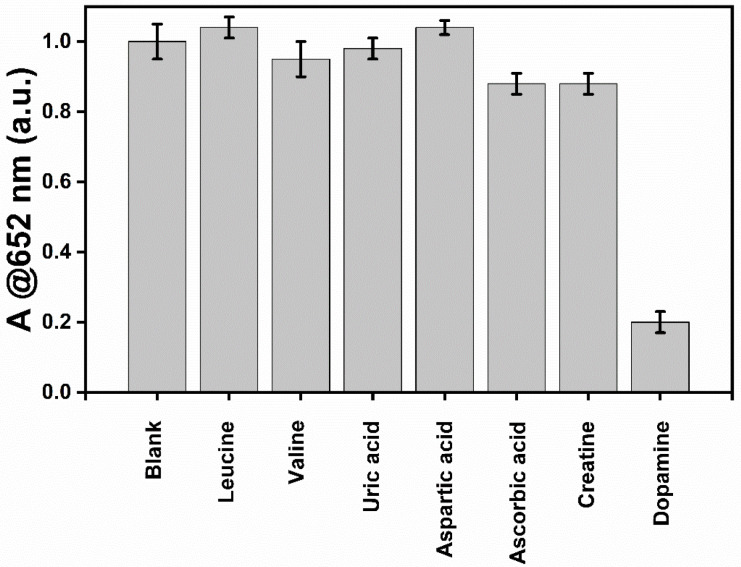
Selectivity of this detection platform for DA assay. From left to right: blank, leucine, valine, uric acid, aspartic acid, ascorbic acid, creatine, and dopamine. Reaction conditions with final concentrations of TMB at 0.1 mM, H_2_O_2_ at 100 mM, AuNPs at 0.72 pM, and dopamine or interferences of 10 µM in 10 mM of sodium acetate buffer pH 5.0 with the total reaction time of 30 min (15 min for the TMB color formation and 15 min for the reaction with DA or interferences).

As shown in Figure 8, the TMB-H_2_O_2_ system shows higher absorbance at 650 nm due to the formation of the blue-colored complex. The absorbance decreases sharply even in the presence of 10 µM of DA, showing that DA inhibits the formation of the blue color of the TMB-β-CD-AuNP system. At the same time, other substances with the same concentration cannot affect the catalytic activity of β-CD-AuNPs compared with the blank sample with a slightly less than 12% decrease in absorbance for the ascorbic acid and creatine. We believe a further understanding of the peroxidase-like activity of β-CD-AuNPs, especially understanding the binding sites of TMB and DA, will further enhance the specificity of the detection of dopamine.

## 3. Materials and Methods

### 3.1. Chemicals and Materials

Gold salt, beta-cyclodextrin, phosphate buffer pH 7.0, acetate buffer pH 5.0, hydrogen peroxide, OPD, DA, valine, uric acid, threonine, aspartic acid, lysine, isoleucine, leucine, cysteine, and creatinine were purchased from Thermo Chemicals, Waltham, MA, USA. HPLC-grade Ultrapure water was used in all experiments. All the chemicals were of analytical grades and used as received without further purification.

### 3.2. Synthesis of β-CD-AuNPs

Before synthesizing the gold nanoparticle solution, all glassware was incubated overnight in aqua regia (mixture of HNO_3_ and HCl in a 3:1 *v*/*v* ratio), washed with tap water, and, finally, washed with distilled, deionized (DI) water. β-CD was used as both a reducing and stabilizing agent. DI water (70 mL) was mixed with a phosphate buffer (10 mL, 0.1 M, pH 7.0) and 0.1 M of HAuCl_4_·3H_2_O (0.2 mL in DI water) in a round bottom flask under vigorous stirring. Then, 10 mM of a β-cyclodextrin (20 mL in DI water) aqueous solution was rapidly added into the mixture with continuous stirring to reduce Au^3+^ species to form gold nanoparticles. The solution changed to red within 5 min of the addition of the beta-cyclodextrin. Once the solution changed to red, it was kept boiling for another 15 min and then cooled to room temperature. The wine-red solution of AuNPs was purified, concentrated, and stored at 4 °C in the refrigerator for further use.

### 3.3. Purification and Concentration of β-CD-AuNPs

Purified and concentrated AuNPs were used throughout the experiment unless stated otherwise. AuNP particles were precipitated by centrifugation for 10 min (10,000 r/min) and re-dispersed in DI water. The precipitation–re-dispersion cycle was repeated twice. The final re-dispersion was performed in a way that the solution is 6× concentrated than the original sample.

### 3.4. Characterization of β-CD-AuNPs

#### 3.4.1. UV–Vis Spectroscopy

The optical properties of β-CD-AuNPs were analyzed using a Cary 50 UV-Vis spectrophotometer (Agilent Technologies, Santa Clara, CA, USA). The absorbance spectra were recorded in the range of 300–800 nm range using a 3 mL quartz cuvette.

#### 3.4.2. Transmission Electron Microscopy (TEM)

The TEM imaging data were acquired with JEOL JEM-1400 TEM operated at 120 kV. The TEM sample was prepared following standard protocol: diluting the AuNP-containing liquid to a proper concentration, ultrasonic bating, vortexing, and applying to holy carbon film-supported 300 mesh Cu grids.

#### 3.4.3. X-Ray Photoelectron Spectroscopy (XPS)

The XPS measurement was performed using a ScientaOmicron ESCA 2SR X-ray Photoelectron Spectroscope System equipped with a flood source charge neutralizer. The Au nanoparticles (AuNPs) containing liquid samples were first dried on an Al foil, and, then, the Al foil covered with dried AuNPs was fixed on the sample stage with double-sided Carbon tape. The sample was then loaded into the load lock and pumped until the vacuum was below 5 × 10^−7^ mBar before it was transferred into the sample analysis chamber. The analysis was carried out with a Mono Al Kα x-ray source (1486.6 eV) at the power of 450 W, and the pressure in the analysis chamber was maintained below 7 × 10^−9^ mBar. A wide region survey scan and high-resolution core level scans of all elements were recorded and calibrated with the C_1s_ 284.8 eV as the reference peak. Pass energy and step size used for the survey scan were 50 eV and 0.1 eV, respectively, while the pass energy and step size used for region scans were 30 eV and 0.05 eV, respectively. The core level spectra were deconvoluted to obtain chemical state information.

#### 3.4.4. Fourier-Transform Infrared Spectroscopy (FTIR)

FTIR analysis was conducted to confirm the functionalization of AuNPs with β-CD. The spectra were recorded using a Brucker model alpha platinum-ATR spectrometer. The spectra were measured from 400 to 4000 cm^−1^ with a 4 cm^−1^ resolution using 32 accumulated scans.

### 3.5. Assessment of Peroxidase-like Activity of β-CD-AuNPs

The peroxidase-like activity of β-CD-AuNPs was evaluated by the classical chromogenic reaction of TMB and OPD in the presence of H_2_O_2_. In a typical assay, 2364 µL of an acetate buffer solution (0.01 M, pH 5.0), 12.0 µL of TMB (20 mM stock in DMSO), 24.0 µL of H_2_O_2_, (1 M stock), and 100 μL of 6× concentrated AuNPs were sequentially added directly in the cuvette. The reaction mixture was well mixed several times, and we let it react for fifteen minutes at room temperature. The absorbance spectra or absorbance at 652 nm was then recorded using a Cary 50 UV-Vis spectrophotometer. For the kinetic assay, measurements were carried out in time course mode by monitoring the absorbance change at 652 nm for TMB. The OPD assay also followed the same procedure except that TMB was replaced by OPD, the typical reaction time was 60 min, and absorbance was monitored at 450 nm.

### 3.6. Dopamine Oxidation Studies

In a typical reaction, 2340 µL of an acetate buffer solution (0.01 M, pH 5.0), 12 μL of dopamine (100 mM), 24.0 μL of H_2_O_2_ (10 M stock), and 100 μL of 6× concentrated β-CD-AuNPs were added directly in the cuvette, mixed, and incubated at room temperature for 60 min. The absorbance spectra or absorbance at 480 nm was then recorded using a Cary 50 UV–Vis spectrophotometer.

### 3.7. Dopamine Assay

In a typical assay, 2364 µL of the acetate buffer solution (0.01 M, pH 5.0), 12 μL of TMB (20 mM), 24.0 μL of H_2_O_2_ (10 M stock), and 100 μL of 6× concentrated β-CD-AuNPs were added directly in the cuvette, mixed, and incubated at room temperature for 15 min. To this, 24 μL of the dopamine solution (10 mM stock) was added and reacted for 15 min more before measuring the absorbances or taking the photographs. The absorbance spectra or absorbance at 480 nm was then recorded using a Cary 50 UV-Vis spectrophotometer.

For the calibration curves, the same procedure is followed by preparing the different stock solutions to match the required concentration. For the selectivity experiments, 24 µL of either dopamine or other interferences are added using 1 mM stocks.

## 4. Conclusions

In conclusion, we explored the peroxidase-like activity of the β-CD-AuNPs in different substances, including TMB and DA. We developed a colorimetric signal-off dopamine sensor based on the competition between dopamine and TMB for possible binding sites at the surface of the β-CD-AuNPs. As a result, the presence of dopamine can be detected even through the naked eye (up to a concentration of 3.75 µM) and using a spectrophotometer (up to a concentration of 1.0 µM) by monitoring the disappearance of the blue color of the oxidized form of TMB in the presence of dopamine. Furthermore, dopamine can also be detected in equal concentrations of common interferences, including ascorbic and uric acid. Further understanding the peroxidase-like activity of β-CD-AuNPs will enhance the sensor’s detection limit and specificity. Compared to the previous peroxidase-mimetic nanozymes, which are limited to a specific analyte, our method could be applied for detecting other analytes due to the presence of cyclodextrin, which can form an inclusion of compounds with a large number of analytes.

## Data Availability

The original contributions presented in this study are included in this article/Supplementary Materials; further inquiries can be directed to the corresponding authors.

## Supplementary Materials

The following supporting information can be downloaded at: https://www.mdpi.com/article/10.3390/molecules30020423/s1. Figure S1. Dependence of the oxidation of TMB by β-CD-AuNPs on pH of (A) plot of absorbances as a function of pH (B)the difference in absorbance between sample (TMB in the presence of H_2_O_2_) and control (TMB in the absence of H_2_O_2_). The concentration of TMB = 0.1 mM and H_2_O_2_ = 1 mM and reaction time of 10 min in 0.01M acetate bufferwith different pH. Figure S2. Dependence of peroxidase-like activity of β-CD-AuNPs on (A) Concentration of AuNPs from 0.18 pM to 1.43 pM and (B) Concentration of TMB from 0.05 mM to 0.4 mM. The concentration of TMB = 0.1 mM and H_2_O_2_ = 10 mM and the reaction time of 10 min in 0.01M acetate buffer. Figure S3. Dependence of peroxidase-like activity of β-CD-AuNPs on (A) Concentration of H_2_O_2_ after 10 min of reaction and (B) reaction time. The concentration of TMB = 0.1 mM and H_2_O_2_ = 10 mM in 0.01M acetate buffer. Table S1. Comparison of the reported artificial peroxidases for the colorimetric detection of DA. References [45,46,47,48,49,50,51] are cited in the supplementary materials.
