# Supplementary material for: Colorimetric Detection of Dopamine Based on Peroxidase-like Activity of β-CD Functionalized AuNPs
Source: Molecules. 2025 Jan 20;30(2):423. doi: 10.3390/molecules30020423 (PMC11767741; doi:10.3390/molecules30020423)
Supplement: Supplementary file 1 [file molecules-30-00423-s001.zip › molecules-3404059-supplementary.pdf]

Supplemental Information

Colorimetric Detection of Dopamine based on Peroxidase-like Activity of β-CD Functionalized AuNPs

Sara Anderson [1], Hamish Shepherd [1], Kiran Boggavarapu [2,*] and Janak Paudyal [1,2,*]

[1] Department of Chemistry and Biochemistry, University of Colorado, Colorado Springs, CO, USA
[2] Department of Chemistry and Physics, McNeese State University, Lake Charles, LA, USA
* Correspondence: kiran@mcneese.edu; jpaudyal@mcneese.edu

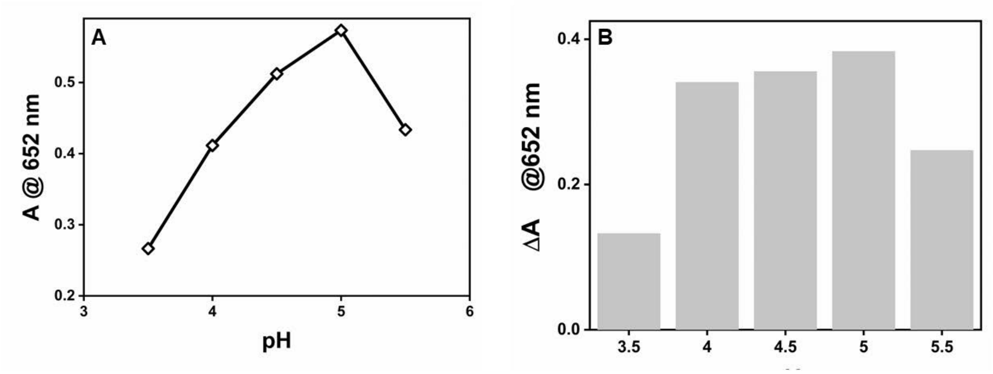

Figure S1. Dependence of the oxidation of TMB by β-CD-AuNPs on pH of (A) plot of absorbances as a function of pH (B) the difference in absorbance between sample (TMB in the presence of H_2O_2) and control (TMB in the absence of H_2O_2). The concentration of TMB = 0.1 mM and H_2O_2 = 1 mM and reaction time of 10 minutes in 0.01M acetate buffer with different pH.

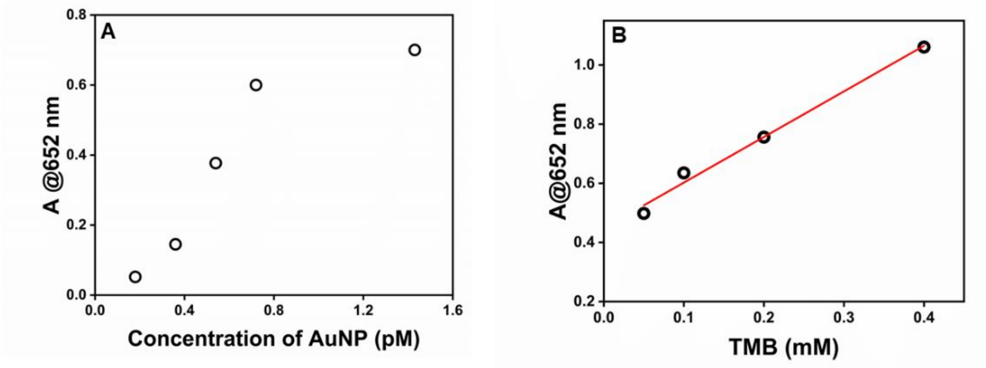

Figure S2. Dependence of peroxidase-like activity of β-CD-AuNPs on (A) Concentration of AuNPs from 0.18 pM to 1.43 pM and (B) Concentration of TMB from 0.05 mM to 0.4 mM. The concentration of TMB = 0.1 mM and H_2O_2 = 10 mM and the reaction time of 10 minutes in 0.01M acetate buffer.

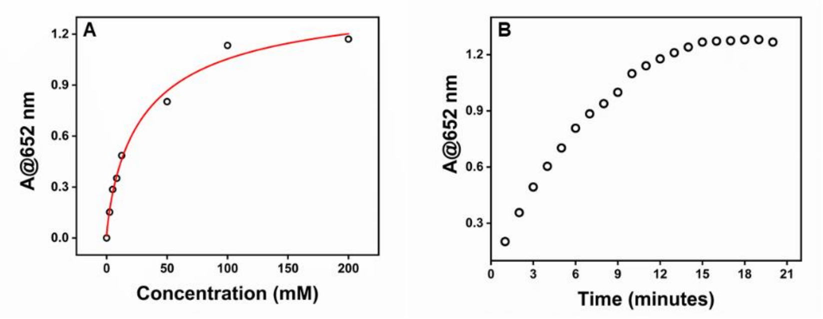

Figure S3. Dependence of peroxidase-like activity of β-CD-AuNPs on (A) Concentration of H_2O_2 after 10 minutes of reaction and (B) reaction time. The concentration of TMB = 0.1 mM and H_2O_2 = 10 mM in 0.01M acetate buffer.

Table S1. Comparison of the reported artificial peroxidases for the colorimetric detection of DA

	**Material**	**Synthesis conditions**	**Linear range (µM)**	**LOD(µM)**	**References**
1.	Cu-BTA-COF	110 °C for 5 days followed by vacuum drying at 60 °C for 24 h	0.2 to 40	0.008	[1]
2.	Cu (PDA)(DMF)	95 °C for 48 h	0-200	-	[2]
3.	CuS-rGO	0.5 hr graphene dispersion and 85 °C for 3 h redox transformation reaction	2-100	0.48	[3]
4.	$Pt/CoSn(OH)_6$	-	5.0 to 60.0	0.76	[4]
5.	$CuFe_2O_4@Cu_9S_8$ @PPy NTs	25°C for 12.0 h	2.0-20.0	-	[5]
6.	Co_3O_4 @NiO NTs	80 °C for 1.0 h	1.0-20.0	0.58	[6]
7.	$Ag_2S@CeO_2$ NPs	25 °C for 5.0 h	0.5-4.0	0.98	[7]
8.	Pt@hBNNS NPs	80 °C for 20.0 h	2.0-55	0.76	[8]
9.	$Pt@CoFe_2O_4$ NPs	60 °C for 6.0 h	20.0-80.0	0.42	[9]
10.	ZIF-67@Co_3O_4 HNCs	25 °C for 24.0 h	4.8-90.0	0.03	[10]
11.	Co_3O_4@NiO	80 ºC for 1 h, followed by 7 hr aging and calcined in air at 500 ºC for 2 h	1–1000	1.21	[11]
12.	β-CD-AuNPs	100°C for 0.5 hr	1.25 to 12.5	1.0	Current work

References

[1] J. Y. Yue, L. P. Song, Y. T. Wang, P. Yang, Y. Ma, and B. Tang, "Fluorescence/Colorimetry/Smartphone Triple-Mode Sensing of Dopamine by a COF-Based Peroxidase-Mimic Platform," *Anal Chem*, vol. 94, no. 41, pp. 14419–14425, Oct. 2022, doi: 10.1021/acs.analchem.2c03179.

[2] J. Wang, Y. Hu, Q. Zhou, L. Hu, W. Fu, and Y. Wang, "Peroxidase-like Activity of Metal-Organic Framework [Cu(PDA)(DMF)] and Its Application for Colorimetric Detection of Dopamine," *ACS Appl Mater Interfaces*, vol. 11, no. 47, pp. 44466–44473, Nov. 2019, doi: 10.1021/acsami.9b17488.

[3] S. Dutta *et al.*, "A Gel-Based Approach to Design Hierarchical CuS Decorated Reduced Graphene Oxide Nanosheets for Enhanced Peroxidase-like Activity Leading to Colorimetric Detection of Dopamine," *Journal of Physical Chemistry C*, vol. 119, no. 41, pp. 23790–23800, Oct. 2015, doi: 10.1021/acs.jpcc.5b08421.

[4] H. Liu *et al.*, "Rapid colorimetric determination of dopamine based on the inhibition of the peroxidase mimicking activity of platinum loaded CoSn(OH)6 nanocubes," *Microchimica Acta*, vol. 186, no. 12, Dec. 2019, doi: 10.1007/s00604-019-3940-5.

[5] Z. Yang, F. Ma, Y. Zhu, S. Chen, C. Wang, and X. Lu, "A facile synthesis of CuFe2O4/Cu9S8/PPy ternary nanotubes as peroxidase mimics for the sensitive colorimetric detection of H2O2 and dopamine," *Dalton Transactions*, vol. 46, no. 34, pp. 11171–11179, Aug. 2017, doi: 10.1039/C7DT02355C.

[6] Y. Zhu, Z. Yang, M. Chi, M. Li, C. Wang, and X. Lu, "Synthesis of hierarchical Co3O4@NiO core-shell nanotubes with a synergistic catalytic activity for peroxidase mimicking and colorimetric detection of dopamine," *Talanta*, vol. 181, pp. 431–439, May 2018, doi: 10.1016/J.TALANTA.2018.01.019.

[7] J. Lian *et al.*, "Multi-layer CeO2-wrapped Ag2S microspheres with enhanced peroxidase-like activity for sensitive detection of dopamine," *Colloids Surf A Physicochem Eng Asp*, vol. 565, pp. 1–7, Mar. 2019, doi: 10.1016/J.COLSURFA.2018.12.047.

[8] M. N. Ivanova *et al.*, "Pt-Decorated Boron Nitride Nanosheets as Artificial Nanozyme for Detection of Dopamine," *ACS Appl Mater Interfaces*, vol. 11, no. 25, pp. 22102–22112, Jun. 2019, doi: 10.1021/acsami.9b04144.

[9] F. He *et al.*, "Pt deposited on magnetic CoFe2O4 nanoparticles: Double enzyme-like activity, catalytic mechanism and fast colorimetric sensing of dopamine," *Microchemical Journal*, vol. 158, p. 105264, Nov. 2020, doi: 10.1016/J.MICROC.2020.105264.

[10] H. Wang, W. Fu, Y. Chen, F. Xue, and G. Shan, "ZIF-67-derived Co3O4 hollow nanocage with efficient peroxidase mimicking characteristic for sensitive colorimetric biosensing of dopamine," *Spectrochim Acta A Mol Biomol Spectrosc*, vol. 246, p. 119006, Feb. 2021, doi: 10.1016/J.SAA.2020.119006.
